# Heparin Therapy Improving Hypoxia in COVID-19 Patients – A Case Series

**DOI:** 10.3389/fphys.2020.573044

**Published:** 2020-10-19

**Authors:** Elnara Marcia Negri, Bruna Mamprim Piloto, Luciana Kato Morinaga, Carlos Viana Poyares Jardim, Shari Anne El-Dash Lamy, Marcelo Alves Ferreira, Elbio Antonio D’Amico, Daniel Deheinzelin

**Affiliations:** ^1^Sirio-Libanes Hospital, São Paulo, Brazil; ^2^Cell Biology Laboratory (LIM 59), Hospital das Clinicas, University of Sao Paulo Medical School, São Paulo, Brazil; ^3^Department of Pulmonary, Heart Institute, University of Sao Paulo Medical School, São Paulo, Brazil; ^4^CriticalCare Unit, A. C. Camargo Cancer Center, São Paulo, Brazil; ^5^Department of Hematology and Hemotherapy, University of Sao Paulo Medical School, São Paulo, Brazil

**Keywords:** COVID-19, respiratory failure, thrombosis, perfusion mismatch, heparin

## Abstract

**Introduction:**

Elevated D-dimer is a predictor of severity and mortality in COVID-19 patients, and heparin use during in-hospital stay has been associated with decreased mortality. COVID-19 patient autopsies have revealed thrombi in the microvasculature, suggesting that hypercoagulability is a prominent feature of organ failure in these patients. Interestingly, in COVID-19, pulmonary compliance is preserved despite severe hypoxemia corroborating the hypothesis that perfusion mismatch may play a significant role in the development of respiratory failure.

**Methods:**

We describe a series of 27 consecutive COVID-19 patients admitted to Sirio-Libanes Hospital in São Paulo-Brazil and treated with heparin in therapeutic doses tailored to clinical severity.

**Results:**

PaO2/FiO2 ratio increased significantly over the 72 h following the start of anticoagulation, from 254(±90) to 325(±80), *p* = 0.013, and 92% of the patients were discharged home within a median time of 11 days. There were no bleeding complications or fatal events.

**Discussion:**

Even though this uncontrolled case series does not offer absolute proof that micro thrombosis in the pulmonary circulation is the underlying mechanism of respiratory failure in COVID-19, patient’s positive response to heparinization contributes to the understanding of the pathophysiological mechanism of the disease and provides valuable information for the treatment of these patients while we await the results of further prospective controlled studies.

## Introduction

Since the beginning of the COVID-19 pandemic, disease severity has been linked to markers of coagulation disturbances such as prothrombin time prolongation, elevated fibrin degradation products, reduced platelet count, and specially to elevated D dimer (Chen G. et al., 2020, Chen T. et al., 2020; Han et al., 2020; Tang et al., 2020; Wang D. et al., 2020; Zhang et al., 2020; Zhou et al., 2020). Higher levels of D dimer and the presence of other coagulation disturbances have been independently associated with development of respiratory failure and death in patients with COVID-19 (Wu et al., 2020). The use of heparin, particularly in those patients with more pronounced elevations of D dimer and in those with elevated sepsis induced coagulopathy (SIC) score, has been associated with a better prognosis (Tang et al., 2020; Wu et al., 2020). Diabetic patients, whose levels of D dimer are greater than those of non-diabetic patients, have also been shown to have a worse prognosis regarding COVID-19 (Guo et al., 2020). Moreover, hypercoagulative features can differentiate severe COVID-19 associated pneumonia from that caused by other viruses (Yin et al., 2020).

Over the last months it has been consistently shown that SARS-Cov-2 causes a cytokine storm, endothelial and epithelial dysfunction, which ultimately lead to the activation of the coagulation cascade, causing thrombotic phenomena (Mehta et al., 2020; Tang et al., 2020; Teuwen et al., 2020; Wu et al., 2020). Similarly to what happens in severe sepsis, the widespread deposition of intravascular clots compromises adequate blood supply, contributing to organ failure (Burzynski et al., 2019).

Disseminated intravascular coagulation (DIC) secondary to severe infection is classically associated with gram-positive or gram-negative bacteria, malaria and haemorrhagic fevers, but other viruses, such as dengue (an hemorrhagic virus), SARS-CoV and MERS-CoV, can also be responsible for systemic activation of intravascular coagulation (de Wit et al., 2016; Giannis et al., 2020).

Furthermore, in contrast to the characteristic stiffening of the lung usually seen in acute respiratory distress syndrome (ARDS), in COVID-19 patients the severe hypoxemia observed is accompanied by near normal pulmonary compliance, especially in early stages (Gattinoni et al., 2020). Autopsy findings from COVID-19 patients show microthrombi in the pulmonary microvasculature (Dolhnikoff et al., 2020; Tian et al., 2020; Yao et al., 2020) suggesting that ventilation-perfusion mismatch due to capillary obstruction could be a pivotal feature in the refractory hypoxemia presented by these patients. The anatomical distribution of this peripheral vascular bed mirrors the predominantly distal and patchy distribution of the radiological infiltrates (Ye et al., 2020).

In one of our first COVID-19 patients we noticed a concomitance of peripheral ischaemia (acro-ischemia) with the onset of respiratory distress, an observation that led us to consider the hypothesis that the normal compliance respiratory failure might actually be due to extensive pulmonary capillary obstruction, and that an intense process of intravascular coagulation might be playing a significant role in hypoxemia and outcome of COVID-19 patients.

The treatment of DIC consists in slowing down the coagulation cascade by using low doses of anticoagulation, alongside vigorous specific treatment of the underlying disorder. We therefore considered adding early heparin therapy to our standard care (Chen G. et al., 2020). The present study is a description of the outcome, particularly regarding oxygenation, of the first 27 COVID-19 patients we treated with anticoagulation in the course of the disease.

## Methods

This study is a case series of 27 consecutive COVID-19 patients seen by our team in Sirio-Libanês Hospital – São Paulo, Brazil, between March 21st and April 12th, 2020. The study was approved by the Sirio-Libanês Hospital Institutional Review Board under the number 3993056 and informed consent was waived.

All patients received initially enoxaparin 0.5 mg/kg SC every 24 h. Patients with a creatinine clearance under 30 mL/min received subcutaneous unfractionated heparin at a dose of 5,000 units every 8 h. If an abrupt decrease in oxygenation or an increase in D Dimer levels was observed, enoxaparin dose was raised to 0.5 mg/kg SC every 12 h and, in the event of thrombotic phenomena or worsening hypoxia, the dose was further increased to 1 mg/kg SC every 12 h. Patients with a BMI (body mass index) of 35 or higher were also considered for the higher dose regimen. Patients in shock or intubated were treated from the beginning with intravenous heparin, targeting an APTT ratio around 1.5 to 2.0 times the normal range. If a patient presented any acute thrombotic event, heparin dosing was increased to obtain an APTT approximately 2.0 to 2.5 times the normal range.

All patients received a 10-day course of azithromycin (500 mg on day 1, then 250 mg daily) (Cramer et al., 2017). Methylprednisolone 40 mg daily was initiated if a worsening in the radiological pattern accompanied by an increase in serum LDH levels was observed. If the patient presented subsequent rise in C-reactive protein, we actively searched for secondary infection and promptly initiated antibiotics.

To evaluate severity of disease during hospital stay, we used the ordinal scale for clinical improvement proposed by the World Health Organization (WHO score): 0. – no clinical or virological evidence of infection; 1. no limitation of activities; 2. limitations of activities; 3. hospitalized, no oxygen therapy; 4. oxygen by mask or nasal prongs; 5. non-invasive ventilation or high-flow oxygen; 6. intubation and mechanical ventilation; 7. ventilation plus additional organ support (pressors, renal replacement therapy, ECMO); 8. death (World Health Organization [WHO], 2020).

## Results

We followed a total of 27 hospitalized patients with a diagnosis of COVID-19, all confirmed by PCR. Seventy percent were male, their mean age was 56 ± 17 years, mean BMI was 28.8 ± 6 kg/m^2^, and comorbidities were present in 67% of them. Individual data from all patients are presented at Table 1. The mean WHO score at admittance was 4.0 ± 1.2 (and the mean maximum score achieved during hospitalization was 4.6 ± 1.6). Entry CT scans showed radiologic infiltrates compromising up to 25% of lung area in 22% of patients, 25–50% of lung area in 48% of patients, and 30% of patients presented infiltrates in over half of lung parenchyma. Symptoms started at an average of 9.6 ± 4.0 days prior to hospitalization, and the anticoagulation protocol was initiated at an average of 3.4 ± 4.0 days after admission. Nineteen patients received methylprednisolone in the course of the disease. Six patients received only the prophylactic dosage of heparin or enoxaparin; three patients started already with enoxaparin 0.5 mg/Kg twice and were kept on this dosage and in 18 patient’s dosages were escalated to either full EV heparin or enoxaparin 1 mg/kg twice a day.

**TABLE 1:** Individual data from all patients included at admission and during evolution.

**Subject**	**Age (years)**	**Gender**	**Comorbi- dities**	**WHO score**	**D-dimer (ng/mL FEU)**	**LDH (U/L)**	Platelet count (*10^3^/mm^3^)	**Antibiotics**	**Corticos- teroids**	**Anticoagu lation**	**In-hospital days**	**Bleeding**	**Macro-thrombosis**	**Outcome**
1	52	M	None	Adm – 3 Max – 4	Adm – 480 Max – 20000	Adm – 271 Max – 271		Yes	Yes	Enoxaparin 2 mg/kg/d	9	No	No	Discharged
2	45	M	None	Adm – 4 Max – 4	Adm – 1360 Max – 780	Adm – 653 Max – 653	Adm – 247 Min – 247 Max – 514	Yes	Yes	Enoxaparin 1 mg/kg/d	4	No	No	Discharged
3	46	F	Breast cancer	Adm – 3 Max – 3	Adm – 561 Max – 561	Adm – 549 Max – 635	Adm – 175 Min – 232 Max – 175	Yes	Yes	Enoxaparin 0.5 mg/kg/d	4	No	No	Discharged
4	46	M	None	Adm – 3 Max – 3	Adm – 1053 Max – 1053	Adm – 532 Max – 532	Adm – 176 Min – 176 Max – 279	Yes	No	Enoxaparin 0.5 mg/kg/d	5	No	No	Discharged
5	79	M	Atrial fibrillation	Adm – 4 Max – 4	Adm – 587 Max – 685	Adm – 540 Max – 626	Adm – 104 Min – 101 Max – 560	Yes	Yes	Enoxaparin 1 mg/kg/d	14	No	No	Discharged
6	66	M	Obesity Hypertension COPD	Adm – 4 Max – 7	Adm – 687 Max – 7519	Adm – 502 Max – 902	Adm – 142 Min – 129 Max – 340	Yes	Yes	IV Heparin	57	No	No	Discharged
7	39	M	None	Adm – 3 Max – 3	Adm – 339 Max – 1228	Adm – 498 Max – 700	Adm – 172 Min – 172 Max – 749	Yes	No	Enoxaparin 1 mg/kg/d	10	No	No	Discharged
8	96	F	COPD Parkinson	Adm – 3 Max – 4	Adm – 525 Max – 1853	Adm – 605 Max – 609	Adm – 204 Min – 167 Max – 254	Yes	Yes	Enoxaparin 1 mg/kg/d	9	No	No	Discharged
9	63	M	Diabetes	Adm – 4 Max – 7	Adm – 644 Max – 6899	Adm – 731 Max – 810	Adm – 155 Min – 155 Max – 474	Yes	Yes	IV Heparin	76	No	No	Discharged
10	76	M	Coronary Heart Disease	Adm – 4 Max – 7	Adm – 1583 Max – 6522	Adm – 805 Max – 805	Adm – 133 Min – 111 Max – 580	Yes	Yes	IV Heparin	142	No	No	Still in hospital
11	68	F	Hypertension Hyperthy- roidism	Adm – 3 Max – 7	Adm – 316 Max – 954	Adm – 436 Max – 648	Adm – 225 Min – 167 Max – 404	Yes	Yes	IV Heparin	24	No	No	Discharged
12	76	F	Atrial fibrillation	Adm – 3 Max – 7	Adm – 1968 Max – > 10000	Adm – 660 Max – 1045	Adm – 158 Min – 144 Max – 419	Yes	Yes	IV Heparin	104	No	VTE	Discharged
13	64	M	Hypertension Diabetes	Adm – 3 Max – 7	Adm – 342 Max – 3014	Adm – 508 Max – 518	Adm – 190 Min – 190 Max – 550	Yes	Yes	IV Heparin	30	No	No	Discharged
14	55	M	Hypertension	Adm – 5 Max – 6	Adm – 679 Max – 3599	Adm – 705 Max – 705	Adm – 295 Min – 295 Max – 460	Yes	Yes	IV Heparin	16	No	No	Discharged
15	66	M	Hypertension	Adm – 3 Max – 3	Adm – 441 Max – 441	Adm – 566 Max – 566	Adm – 224 Min – 224 Max – 285	Yes	No	Enoxaparin 0.5 mg/kg/d	3	No	No	Discharged
16	45	F	None	Adm – 3 Max – 3	Adm – 510 Max – 843	Adm – 461 Max – 461	Adm – 279 Min – 279 Max – 334	Yes	No	Enoxaparin 0.5 mg/kg/d	5	No	No	Discharged
17	53	F	Hypertension	Adm – 3 Max – 4	Adm – 395 Max – 406	Adm – 743 Max – 1034	Adm – 249 Min – 249 Max – 706	Yes	Yes	Enoxaparin 1 mg/kg/d	8	No	No	Discharged
18	35	M	Obesity Asthma	Adm – 3 Max – 3	Adm – 438 Max – 438	Adm – 520 Max – 599	Adm – 235 Min – 205 Max – 266	Yes	No	Enoxaparin 0.5 mg/kg/d	3	No	No	Discharged
19	52	M	Tobacco dependence	Adm – 3 Max – 4	Adm – 505 Max – 912	Adm – 368 Max – 830	Adm – 167 Min – 140 Max – 423	Yes	No	Enoxaparin 0.5 mg/kg/d	15	No	No	Discharged
20	67	F	Treated lymphoma	Adm – 3 Max – 4	Adm – 823 Max – 1234	Adm – 636 Max – 888	Adm – 125 Min – 105 Max – 325	Yes	Yes	Enoxaparin 1 mg/kg/d	12	No	No	Discharged
21	32	M	None	Adm – 3 Max – 4	Adm – 416 Max – 416	Adm – 408 Max – 797	Adm – 278 Min – 274 Max – 515	Yes	Yes	Enoxaparin 2 mg/kg/d	13	No	No	Discharged
22	66	M	Hypertension	Adm – 3 Max – 7	Adm – 720 Max – 4833	Adm – 611 Max – 618	Adm – 271 Min – 256 Max – 498	Yes	Yes	IV Heparin	22	No	No	Discharged
23	22	M	None	Adm – 3 Max – 3	Adm – 245 Max – 362	Adm – 520 Max – 970	Adm – 249 Min – 249 Max – 290	Yes	No	Enoxaparin 1 mg/kg/d	3	No	No	Discharged
24	42	F	None	Adm – 4 Max – 4	Adm – 644 Max – 1242	Adm – 571 Max – 716	Adm – 263 Min – 263 Max – 408	Yes	Yes	Enoxaparin 1 mg/kg/d	6	No	No	Discharged
25	65	M	Obesity	Adm – 4 Max – 4	Adm – 569 Max – 1346	Adm – 571 Max – 571	Adm – 186 Min – 179 Max – 265	Yes	No	Enoxaparin 1 mg/kg/d		No	No	Transferred
26	79	M	Cerebro- vascular disease	Adm – 3 Max – 4	Adm – 1449 Max – 1686	Adm – 535 Max – 678	Adm – 267 Min – 217 Max – 318	Yes	Yes	Enoxaparin 1 mg/kg/d	12	No	No	Discharged
27	35	M	None	Adm – 4 Max – 5	Adm – 555 Max – 605	Adm – 630 Max – 709	Adm – 134 Min – 134 Max – 331	Yes	Yes	Enoxaparin 2 mg/kg/d	9	No	No	Discharged

As of August 11th, of the 27 consecutive patients, 25(92%) were discharged from hospital after an median of 11 days. One patient was transferred to another hospital on the 4th day and lost follow-up. Nine patients (33%) were admitted to ICU, 8 (89%) of which have already been discharged to the ward after a median time of 44 days. Eight patients (30%) required intubation, and seven patients have already been successfully weaned after a median time of 11,5 days of mechanical ventilation. One patient is still under mechanical ventilation and required a tracheostomy. This patient had an infected sacral pressure ulcer and required multiple surgical procedures.

Interestingly enough, rotational thromboelastometry (ROTEM) performed in four patients, showed an increase in α-angle, amplitude 10 min after clotting time (A10) and maximum clot firmness (MCF) pointing to a persistent hypercoagulability state, despite their ongoing heparin use.

**FIGURE 1:**
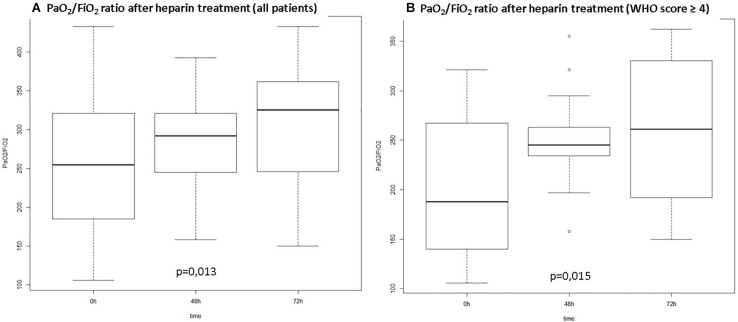
PO_2_/FiO_2_ ratio over time from start of anticoagulation. **(A)** All patients included; **(B)** Patients with WHO score ≥4 at hospital admission (WHO Score: ordinal scale for clinical improvement proposed by the World Health Organization: 0. – no clinical or virological evidence of infection; 1. no limitation of activities; 2. limitations of activities; 3. hospitalized, no oxygen therapy; 4. oxygen by mask or nasal prongs; 5. non-invasive ventilation or high-flow oxygen; 6. intubation and mechanical ventilation; 7. ventilation plus additional organ support – pressors, renal replacement therapy, ECMO; 8. Death).

Figure 1 depicts the gradual improvement in PaO_2_/FiO_2_ ratio along the first 72 h in relation to pre-anticoagulation values. Analysis was conducted for the whole series (A) and considering only patients with moderate to severe disease (B) according to the WHO score (*p* < 0.02 for both groups). For non-mechanically ventilated patients PaO_2_/FiO_2_ ratio was calculated according to mask or nasal catheter oxygen flow and oxymetry (Lobete et al., 2013).

We observed no deaths due to any cause or haemorrhagic complications due to anticoagulation during the study period. Moreover, after three months, all but one patient were discharged home without supplementary oxygen.

## Discussion

Our results suggest the important role of hypercoagulative state and microthrombosis as the main mechanisms of organ failure in COVID-19 and the potential response to early anticoagulation therapy.

The significant improvement in oxygen exchange and clinical symptoms observed in these COVID-19 patients, in response to the anticoagulation, points to a potential role for systematic use of heparin in the treatment of such patients. The high incidence of thrombotic events that has been reported in COVID-19 patients (Klok et al., 2020), confirmed more recently, even in the presence of usual heparin prophylaxis (Middeldorp et al., 2020), as well as the fact that similar observations were reported in the other recent coronavirus outbreaks (de Wit et al., 2016; Giannis et al., 2020), further corroborate with this line of reasoning. This is not surprising, as severe cases of COVID-19 meet the laboratory criteria of DIC (Tang et al., 2020; Wu et al., 2020) of thrombotic pattern, in which fibrinogen does not drop and prothrombotic phenomena override the haemorrhagic ones (Wada et al., 2014). Moreover, specifically in patients with respiratory insufficiency caused by COVID-19 under mechanical ventilation, increased d-dimer, abnormal thromboelastography and high levels of fibrinogen point to a hypercoagulative status (Ranucci et al., 2020; Wright et al., 2020). Markers of hypercoagulability has been shown to be independent predictors of increased oxygen requirements in patients with COVID-19 (Rauch et al., 2020).

Thromboelastography showing a pattern of hypercoagulability despite the use of heparin during the course of viral diseases has been previously reported (Wilson et al., 2016). In fact, many viruses known to induce a state of hypercoagulability (Subramaniam and Scharrer, 2018) have a similar pattern of disease, including the timeframe of clinical manifestations (Gai et al., 2012), suggesting a common pattern of response.

Multiple phenomena are involved in the hypercoagulative status in COVID 19: the extensive denudation of epithelial and endothelial spaces causing a massive exposure of tissue factor, production of Von Willeband factor, platelet activation, netosis and pyroptosis, have been described as promoters of extensive microcirculation thrombosis in the severe cases of this disease (Wada et al., 2014; Teuwen et al., 2020). It has been shown that in patients with COVID-19, NETs increased with intubation or death as outcome and were inversely correlated with PaO2/FiO2 (Brinkmann et al., 2004; Middleton et al., 2020). Many autopsy findings confirm this pathophysiological rationale, showing a large amount of microthrombosis as well venous and arterial thrombosis in deceased patients (Dolhnikoff et al., 2020; Yao et al., 2020). Ultrastructural findings also corroborate the endothelial and epithelial destruction in multiple organs (Ackermann et al., 2020). More recently heparin treatment has been pointed to decrease mortality in severe COVID-19 (Ayerbe et al., 2020).

The PaO_2_/FiO_2_ ratio improvement observed in our patients after starting heparin is in agreement with the idea of a significant perfusion component explaining the mechanism of respiratory failure with the distinct pattern of marked hypoxia and preserved lung compliance that characterizes severe COVID-19 patients. It has been argued that this could be secondary to the loss of lung perfusion regulation and hypoxic vasoconstriction (Tian et al., 2020), but the clinical response to heparin rather suggests hypoxia due to extensive clogging of pulmonary microcirculation. HRCT studies have shown a consistent reduction in pulmonary blood volume in COVID-19 patients compared to healthy controls, particularly in vessels smaller than 5 mm, again pointing to microthrombi as a cause for hypoxia (Lins et al., 2020). Using electrical impedance tomography, it has been shown that dead space fraction was much more relevant than the shunt fraction as an explanation for the gas exchange derangement observed in the course of disease (Mauri et al., 2020). Moreover when a diagnosis of pulmonary embolism is made in patients with COVID-19, the phenotype is different than embolism in other patients, since it occurs only in areas already affected by the virus, with a lower thrombus load and lower prevalence of proximal embolism of main arteries (van Dam et al., 2020). Interestingly the use of tissue Plasminogen Activator (tPA) has been shown to promote a non-sustained elevation of PaO2/FiO2 ratio (Wang J. et al., 2020). In our opinion, given the marked hypercoagulability seen in these patients – and again in accordance with the autopsy findings – judicious tailoring of heparin doses is needed to prevent capillary reocclusion while avoiding the risks of bleeding complications.

The fact that this is a retrospective study without a control arm does not yet allow us to definitively conclude that heparin in tailored doses should be systematically employed in all COVID-19 patients. Nonetheless, our findings in this early group of patients certainly provide food for thought and perhaps a rationale to justify using a readily available and well-known drug such as heparin, even in larger doses than previously recommended to ameliorate the dim prognosis of such sick patients while we await the more solid data on this subject, as suggested recently (Iba et al., 2020).

## Data Availability Statement

The raw data supporting the conclusions of this article will be made available by the authors, without undue reservation.

## Ethics Statement

The studies involving human participants were reviewed and approved by the Hospital Sirio-Libanes Institutional Review Board – Number of approval: 3.993.056. Written informed consent for participation was not required for this study in accordance with the national legislation and the institutional requirements.

## Author Contributions

All authors contributed equally on this manuscript.

## Conflict of Interest

The authors declare that the research was conducted in the absence of any commercial or financial relationships that could be construed as a potential conflict of interest.
